# Pharmaceutical waste management through microbial bioremediation

**DOI:** 10.1007/s10661-025-13912-y

**Published:** 2025-03-31

**Authors:** Kishore Srinivasan, Raghu Chandrashekar Hariharapura, Subrahmanyam Volety Mallikarjuna

**Affiliations:** Department of Pharmaceutical Biotechnology, Manipal College of Pharmaceutical Sciences, Manipal Academy of Higher Education, Manipal, Karnataka India

**Keywords:** Pharmaceutical, Bioremediation, Microorganisms, Green pharmacy

## Abstract

Pharmaceuticals play a significant role in enhancing the quality of life. However, pharmaceutical products (PPs) manufacturing presents challenges, particularly in terms of waste generated, posing a risk to the ecosystem. Existing physical and chemical remediation methods are expensive and are not directly applicable for pharmaceutical remediation. Bioremediation using various microbial consortia has the potential to become a cost-effective solution when applied optimally. This review highlights the various pharmaceutical products, their occurrence in the environment, and their associated health risks. Further, various microorganisms employed in the bioremediation process and the techniques utilized to degrade diverse categories of pharmaceutical pollutants are discussed. Finally, the review highlights the limitations of using bioremediation for treating pharmaceutical waste and discusses alternative sustainable green pharmacy approaches to reduce the impact of pharmaceutical contaminants on the environment.

## Introduction

Pharmaceutical products (PPs) have become indispensable in advancing physiological well-being and therapeutic efficacy in living organism. However, the production and usage of various PPs have surged massively in recent times due to shifts in global health dynamics and medical demands. This increase has contributed to products are increasingly posing risks to health and environmental hazards due to inadequate disposal and inefficient treatment processes (Ashiwaju et al., 2023). Significant amounts, ranging from microgram to even picogram levels, are detected in groundwater, topsoil, and the atmosphere. A study reported the presence of nearly 713 active pharmaceutical compounds and their transformation products of commonly used drugs in surface, ground, and drinking water in many countries (aus der Beek et al., 2016). The persistence of these pharmaceutical pollutants has been associated with various toxicological impacts on biological systems. Treating these pharmaceutical compounds poses challenges due to their toxicity, chemical stability, and concentration (Oberoi et al., 2019). Various physical and chemical remediation methods are available for environmental treatment. Physical methods include filtration, coagulation/flocculation, adsorption (e.g., activated carbon, biochar, zeolites), membrane technologies (e.g., reverse osmosis, nanofiltration, ultrafiltration), electrodialysis, and advanced sedimentation while chemical methods involve ozonation, hydrogen peroxide oxidation, plasma-based oxidation, photocatalysis (e.g., TiO₂-based), chlorination, Fenton reaction, and electrochemical oxidation (Adedeji et al., 2022; Saravanan et al., 2021). However, these methods are not directly applicable for remediating pharmaceutical contaminants as they are costly, environmentally harmful, and may lack effectiveness (Chhaya et al., 2020). An alternative approach could be the bioremediation technique that has been efficiently used in treating petrochemical contaminants over decades (Chhaya et al., 2020). This review article emphasizes the different pharmaceutical contaminants present in the environment and their harmful effects. In addition, the article discusses the different bioremediation techniques and microorganisms employed in degrading these contaminants. Finally, the article assesses the limitations of microbial bioremediation in pharmaceutical waste treatment and explores the sustainable green pharmacy approach as a potential alternative.

## Pharmaceutical contaminants and their effects

The use of PPs has significantly increased globally over the years, with antibiotic consumption being a major concern due to the development of antimicrobial resistance (Browne et al., 2021; Leonard et al., 2022; Frascaroli et al., 2021). Once administered, the PPs exert their therapeutic effect before being excreted via urine or feces, eventually reaching the sewage treatment plants (STPs) either in their original form or as metabolite substances (Ashiwaju et al., 2023). While STPs attempt to treat this waste, some PP residues are still discharged into water bodies, where sunlight may partially degrade them through UV exposure. However, studies suggest that many common PPs are resistant to degradation and can persist in the environment, sometimes appearing as pollutants in drinking water (Kawabata et al., 2013; Kounaris Fuziki et al., 2023). The degree of pharmaceutical degradation differs significantly based on the stability of the active components when exposed to atmosphere, light, water, and microorganisms.

In the last few decades, the presence of antibiotics, hormones, β-blockers, lipid-regulators, non-steroidal anti-inflammatory drugs (NSAIDs), and other drugs in wastewater treatment plants (WWTPs) and aquatic reservoirs has drawn considerable attention due to their potential adverse effects on the ecosystem. Antibiotics are often used in the treatment of bacterial infections while NSAIDS are used for their anti-inflammatory, antipyretic, and analgesic effects to treat acute/chronic pain and fever. Hormones are prescribed for physiological processes such as stress responses, sexual maturity, osmoregulation, growth promotion, etc. β-blockers, and lipid-regulators are used to manage various cardiovascular conditions such as blood pressure, stroke, coronary artery disease, and heart attack (Letsoalo et al., 2023; Samal et al., 2022). Each drug class introduces specific environmental and health risks due to its distinct chemical composition and biological activity.

**Fig. 1:**
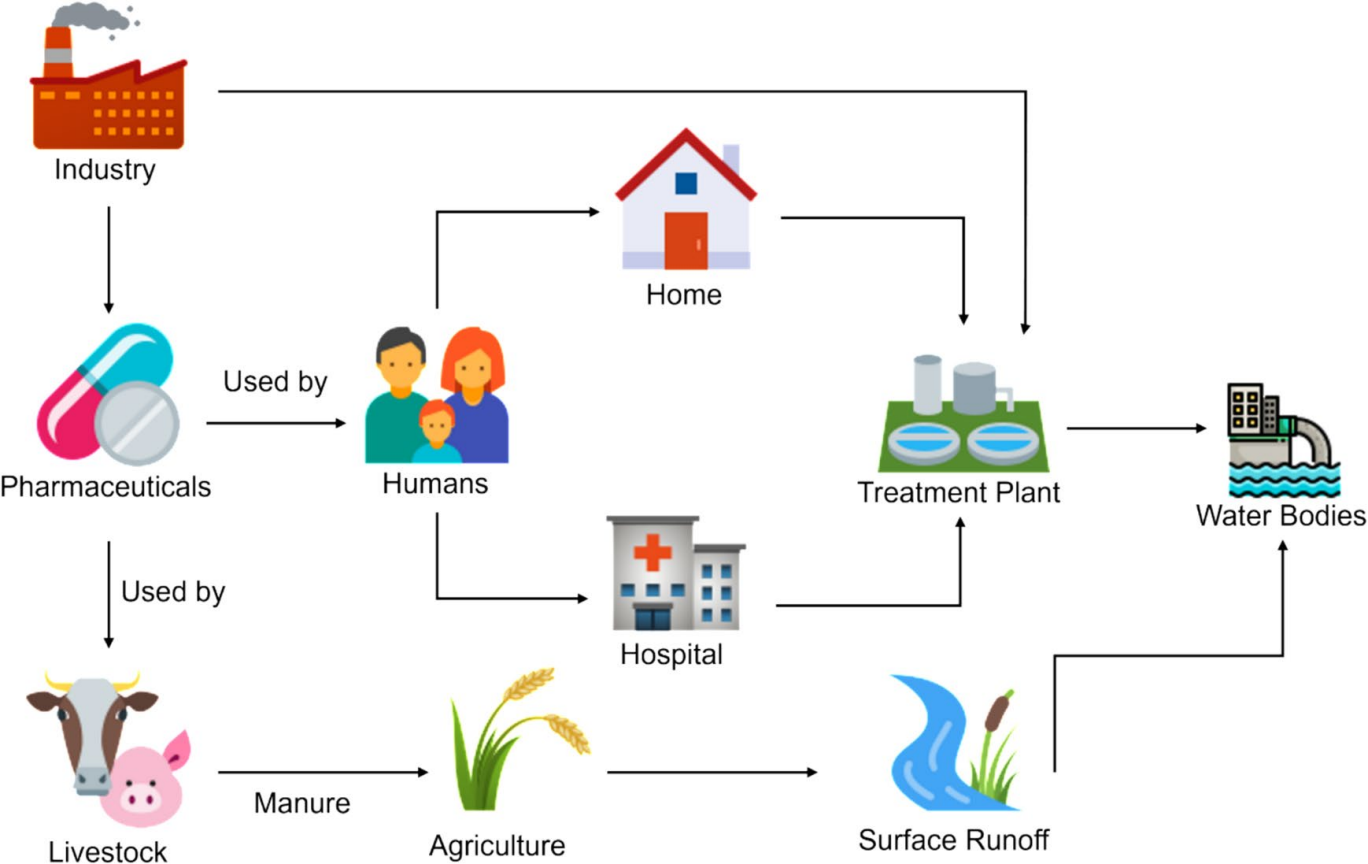
Routes of pharmaceutical discharge into water bodies (the image was created using icons from Icons8; https://icons8.com)

Pharmaceuticals are also termed “pseudo-persistent contaminants” because they are constantly released into the environment through various pathways, including discharges from untreated sewage, wastewater treatment plants, agriculture fields, animal excrement, landfill leachates from household and hospital solid wastes, and unused or expired medicine disposed through landfills and sewage systems (Khasawneh & Palaniandy, 2021). A pictorial representation of the same is provided in Fig. 1. A recent study by Wilkinson and his group analyzed river samples from 104 countries and reported the presence of 61 different pharmaceutical ingredients, with the highest concentrations found in South Asia, Saharan Africa, and South America. Among the various pharmaceutical ingredients, metformin, carbamazepine, and caffeine were detected in higher concentrations (Wilkinson et al., 2022). The diverse properties of the pharmaceuticals such as high polarity, lipophilicity, volatility, persistence, and adsorption can impede their removal during WWTP processes, often resulting in accumulation and toxicity within ecosystems (Khasawneh & Palaniandy, 2021). For instance, diclofenac and its metabolites can interact synergistically with other pollutants resulting in high-risk (Matejczyk et al., 2020). A study by Foster et al. (2010) demonstrated the impact of fluoxetine on the metabolism and gene expression of *Rana pipiens*, resulting in a negative impact on growth, development, and survival. The presence of paracetamol in the aquatic environment led to genotoxicity in the gills and kidneys of *Rhamdia quelen* catfish (Perussolo et al., 2019). Leanardo et al. (2022) highlighted the potential risk caused to human health due to the presence of pharmaceutical contaminants. Another study reported adverse effects such as malformations and mortality in the embryos and apoptosis of larvae of zebrafish caused by NSAIDs (Bereketoglu et al., 2020). Apart from this, the anticancer drugs have the potential to cause chronic genotoxicity (Novak et al., 2017). Addressing these health issues require approaches beyond physical and chemical treatment methods to effectively mitigate pharmaceutical contaminants in the environment.

## Bioremediation of pharmaceutical contaminants

**Fig. 2:**
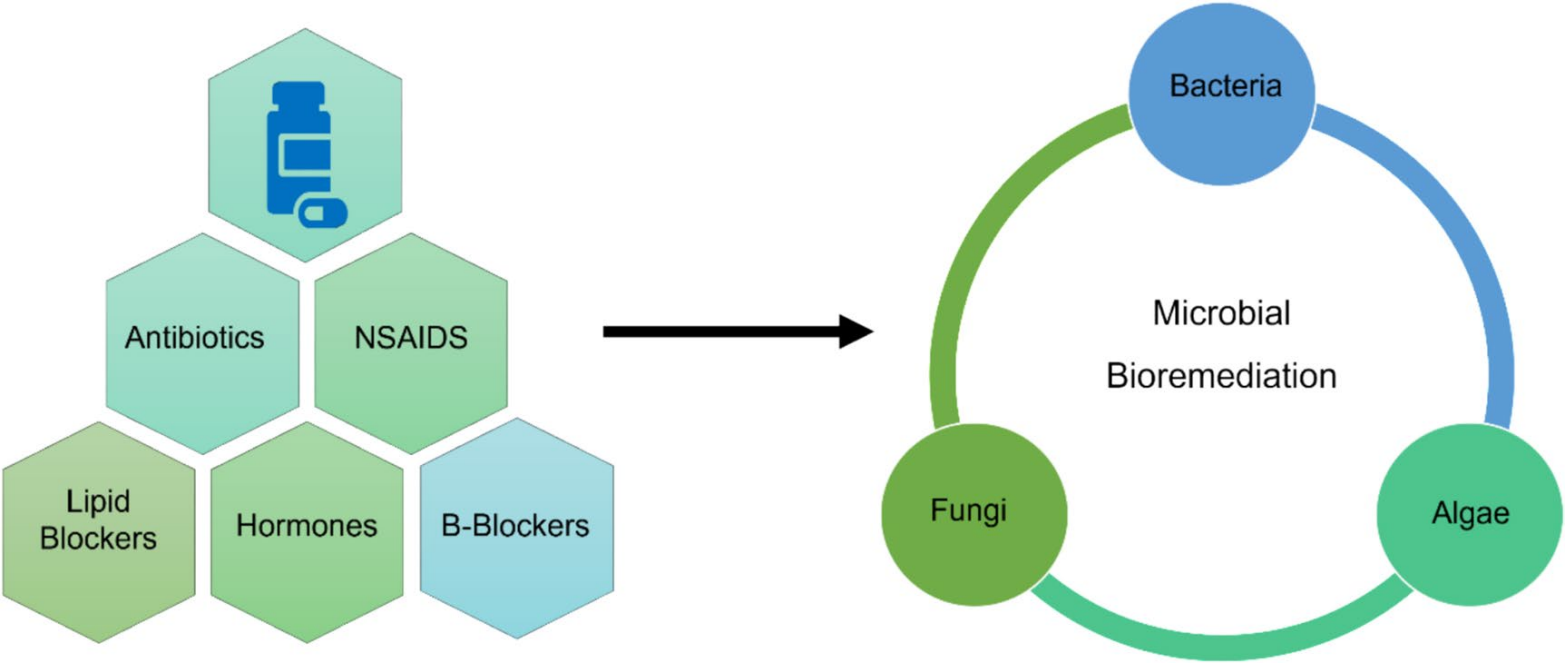
The microorganism used in the remediation of pharmaceutical drugs

To address the challenges posed by pharmaceutical pollutants, bioremediation offers a promising solution. The process involves utilizing diverse microorganisms to detoxify, reduce, degrade, mineralize, or transform unstable or toxic pollutants into non-toxic and stable forms. The wide range of microorganisms used in this technique includes bacteria, algae, fungi, etc., which can be further classified into indigenous (already present in the polluted environment) and nonindigenous (introduced into the polluted environment). Figure 2 represents the microorganism used in the remediation of pharmaceutical drugs. Compared to the physical and chemical methods employed to remediate environmental pollutants, the bioremediation method is cost-effective, environment-friendly, and more sustainable. It is categorized into ex situ and in situ, each with specific benefits and limitations.

### Ex situ bioremediation

In ex situ bioremediation, the pollutants are removed from the contamination site and transported to another location for treatment. Several criteria such as geographical location, degree of pollution, pollutant type, and treatment cost are considered while choosing this technique. Biopile, windrows, land farming, and bioreactors are the techniques that fall under this category (Azubuike et al., 2016). Biopile is an approach where the soil is excavated and piled, which is further aerated and supplied with nutrients to enrich the activity of microbes. The main advantage is that it is cost-effective and feasible for different soil types. This technique has been widely used to eliminate hydrocarbon-based pollutants. Besides, a recent study has demonstrated the removal of pharmaceutical wastes using this approach. The team reported the potential of the fungus *Trametes versicolor* in eliminating 2 out of 15 hydrophobic pharmaceutical drugs (Llorens-Blanch et al., 2018). On the contrary, windrows-based bioremediation requires contaminated soil to be piled in long rows with periodic turnaround and supply of water resulting in increased aeration and nutrient supply. Dalahmeh et al. (2022) have studied the fate of pharmaceutical products such as hormones, antibiotics, and pharmaceutically active compounds in open-air storage of sewage sludge windrow for 12 months. It was observed that composting the windrow caused a reduction of hormones and antibiotics below detectable levels while the active components were reduced by 95% of the initial concentration.

Land farming is the simplest ex situ bioremediation concerning cost and equipment requirements. In this technique, the polluted soil is extracted and spread over a bed prepared for biological degradation. Although considered an ex situ technique, it can also be regarded as an in situ technique based on the site of treatment. Despite several advantages such as low cost, simple design, regulatory compliance, flexibility, and potential for large-volume treatment, certain limitations do occur that include space requirements, environmental sensitivity, excavation costs, limited efficiency for inorganic pollutants, and unsuitability for toxic volatiles (Azubuike et al., 2016).

Bioreactors used as another ex situ bioremediation technique are vessels where the biological reactions occur to produce specific products. Different operating modes, such as batch mode, fed-batch mode, sequencing batch mode, and continuous mode are employed based on the nature of the pollutant. Advantages of utilizing bioreactors include control over process parameters, enhanced biological reactions, versatility, bioaugmentation and stimulation, monitoring of microbial population dynamics, and the use of genetically modified microorganisms (GEM). Different bioreactors such as packed bed reactors, slurry phase reactors, sequence batch reactors, membrane bioreactors, etc. are being utilized in the remediation of various contaminants. Among these, membrane bioreactors have been widely used in effective pharmaceutical bioremediation. For instance, a full-scale membrane bioreactor system was used to remove pharmaceutical pollutants such as diclofenac and carbamazepine (Beier et al., 2011). Besides, photo-oxidation along with membrane bioreactor degraded pharmaceutical active components (Joannis‐Cassan et al., 2021). Another study reported that a membrane bioreactor in integration with an electrochemical advanced oxidation process (AOPs) significantly increased the biodegradability of pollutants in the wastewater. Additionally, enhanced AOP membrane bioreactor processes have been reported to be efficient in removing more than 90% of pharmaceuticals that include ibuprofen, bezafibrate, and carbamazepine under 40 h hydraulic retention time and 400% reflux mixed liquid ratio (Gharibian & Hazrati, 2022).

### In situ bioremediation

In situ bioremediation is a technique where the pollutants are cleaned at the original site of creation. As it eliminates the need for exaction, this technique is cost-effective compared to ex-situ techniques. However, concerns such as the cost of design and installation of equipment do exist. Various factors including nutrient availability, moisture content, temperature, and pH play a pivotal role in achieving successful in-situ bioremediation. Techniques under in-situ bioremediation include bioventing, bioslurping, and biosparging (Azubuike et al., 2016). Bioventing is a technique in which oxygen is delivered to an unsaturated zone through controlled airflow to increase the activity of indigenous microbes. Additionally, nutrients and moisture are supplied to further enhance the bioremediation process. Biosparging is a technique similar to bioventing in which air is injected into the saturated zone. However, the efficiency is determined based on soil permeability and pollutant biodegradability. On the other hand, several techniques such as bioventing, vacuum-assisted pumping, and soil vapor extraction are combined in bioslurping to achieve the remediation of soil and groundwater contaminants (Lakhani et al., 2022). The sites contaminated with chlorinated solvents, heavy metals, and hydrocarbons are remediated using these in situ approaches. However, its application in the remediation of pharmaceutical contaminants is still not reported which presents scope for further research. While various bioremediation methods for treating contaminated soil and water have been outlined, the following section will emphasize the diverse microorganisms used for remediating water bodies impacted by pharmaceutical pollutants.

## Microorganisms used in bioremediation

Recently, using microorganisms for the remediation of pharmaceutical contaminants has drastically increased. Microorganisms such as bacteria, algae, and fungi are being used in this process. The different microbial species utilized for the bioremediation of pharmaceutical drugs are listed in Table 1.

**Table 1:** Microorganisms involved in the biodegradation of pharmaceutical compounds

Drug class	Drugs	Microorganisms	Species	References
Antibiotics	Ciprofloxacin	Bacteria	*Labrys portucalensis F11*, *Thermus* species* strain C419*	(Amorim et al., 2014; Pan et al., 2018)
	Norfloxacin	Bacteria	*Labrys portucalensis F11*	(Amorim et al., 2014)
	Sulfamethazine	Bacteria	*Geobacillus* species* strain S-07*	(Pan et al., 2017)
	Sulfamethoxazole	Bacteria	*Ochrobactrum species SA1*, *Gordonia species SCD14*,* Labrys species SC11*	(Mulla et al., 2018)
		Fungi	*Trametes versicolor*, *Bjerkandera species R1*, *Bjerkandera adusta*, *Phanerochaete chrysosporium*	(Alharbi et al., 2019; Rodarte-Morales et al., 2011)
	Moxifloxacin	Bacteria	*Labrys portucalensis F11*	(Carvalho et al., 2016)
	Clarithromycin	Fungi	*Trichoderma harzianum*, *Pleurotus ostreatus*	(Buchicchio et al., 2016)
	Trimethoprim	Fungi	*Trametes versicolor*	(Alharbi et al., 2019)
	Cefradine	Algae	*Chlorella pyrenoidosa*	(Du et al., 2015)
	Levofloxacin	Algae	*Scenedesmus obliquus*	(Xiong et al., 2017)
	Ofloxacin	Bacteria	*Labrys portucalensis F11*	(Amorim et al., 2014)
NSAIDS	Ibuprofen	Bacteria	*Comamonas aquatica*, *Bacillus* species, *Bacillus thuringiensis B1*	(Fortunato et al., 2016; Marchlewicz et al., 2016)
		Fungi	*Aspergillus niger*, *Trametes polyzona*, *Trichoderma longibrachiatum*, *Rhizopus microspores*, *Mucor circinelloides*, *Trametes versicolor*, *Phanerochaete chrysoporium*, *Irpex lacteus*, *Ganoderma lucidum*, *Bjerkandera species R1*, *Bjerkandera adusta*	(Kasonga et al., 2020; Marco-Urrea et al., 2009; Rodarte-Morales et al., 2011, 2012)
		Algae	*Spirogyra* species	(Garcia-Rodríguez et al., 2015)
	Diclofenac	Bacteria	*Labrys portucalensis F11*, *Brevibacterium species D4*	(Bessa, Moreira, Murgolo, et al., 2017; Bessa, Moreira, Tiritan, et al., 2017a, b; Moreira et al., 2018)
		Fungi	*Trametes versicolor*, *Aspergillus niger*, *Trametes polyzona*, *Trichoderma longibrachiatum*, *Rhizopus microspores*, *Mucor circinelloides*, *Ganoderma lucidum*, *Phanerochaete chrysoporium*, *Bjerkandera species R1*, *Bjerkandera adusta*	(Alharbi et al., 2019; Kasonga et al., 2020; Mohd Hanafiah et al., 2024; Rodarte-Morales et al., 2011, 2012)
	Naproxen	Bacteria	*Bacillus thuringiensis*	(Marchlewicz et al., 2016)
		Fungi	*Bjerkandera speciesR1*, *Bjerkandera adusta*, *Phanerochaete chrysosporium*, *Trametes versicolor*	(Rodarte-Morales et al., 2011, 2012; Rodríguez-Rodríguez et al., 2010)
	Paracetamol (Acetomenophen)	Bacteria	*Stenotrophomonas* species, *Pseudomonas* species	(Zhang et al., 2013)
		Fungi	*Trametes hirsuta*	(Hachi et al., 2017)
		Algae	*Chlorella sorokiniana*, *Spirogyra* species	(Escapa et al., 2017; Garcia-Rodríguez et al., 2015)
Hormones	Ethinylestradiol	Algae	*Chlorella PY-ZU1*, *Spirogyra* species	(Cheng et al., 2018; Garcia-Rodríguez et al., 2015)
β-Blockers	Propranolol	Fungi	*Ganoderma lucidum*	(Mohd Hanafiah et al., 2024)
		Algae	*Spirogyra* species	(Garcia-Rodríguez et al., 2015)
Lipid regulators	Clofibric Acid	Fungi	*Trametes versicolor*	(Marco-Urrea et al., 2009)
Antidepressants	Citalopram	Fungi	*Ganoderma lucidum*, *Bjerkandera* *species R1*, *Bjerkandera adusta*, and *Phanerochaete chrysosporium*	(Mohd Hanafiah et al., 2024; Rodarte-Morales et al., 2011)
	Fluoxetine	Fungi	*Bjerkandera species R1*, *Bjerkandera adusta*, *Phanerochaete chrysosporium*	(Rodarte-Morales et al., 2011)
Anticonvulsants	Carbamazepine	Bacteria	*Labrys portucalensis F11*, Starkeya *species C11*, Rhizobium *species C12*	(Bessa et al., 2019; Bessa, Moreira, Murgolo, et al., 2017; Bessa, Moreira, Tiritan, et al., 2017a, b)
		Fungi	*Trametes versicolor*, *Trichoderma harzianum*, *Pleurotus ostreatus*, *Trametes hirsute*, *Aspergillus niger*, *Trametes polyzona*, *Trichoderma longibrachiatum*, *Rhizopus microspores*, *Mucor circinelloides*, *Ganoderma lucidum*, *Bjerkandera species R1*, *Bjerkandera adusta*, *Phanerochaete chrysosporium*	(Alharbi et al., 2019; Buchicchio et al., 2016; Hachi et al., 2017; Kasonga et al., 2020; Marco-Urrea et al., 2009; Rodarte-Morales et al., 2011; Rodríguez-Rodríguez et al., 2010)
Antimicrobial	Triclosan	Algae	*Nannochloris* species	(Bai & Acharya, 2016)
Cardiovascular agents	Valsartan	Fungi	*Ganoderma lucidum*	(Mohd Hanafiah et al., 2024)
Benzodiazepines	Diazepam	Fungi	*Bjerkandera species R1*, *Bjerkandera adusta*, *Phanerochaete chrysosporium*	(Rodarte-Morales et al., 2011)

### Bacteria

Bacteria are widely utilized in the process of pharmaceutical bioremediation. Studies have employed bacterial cultures that function under both aerobic and anaerobic conditions. Several aerobic bacterial species such as *Bacillus*, *Corynebacterium*, *Arthrobacter*, *Alcaligenes*, *Pseudomonas*, *Mycobacterium*, *Nitrosomonas*, *Achromobacter*, *Xanthobacter*, *Flavobacterium*, etc. and anaerobic bacterial species such as *Clostridium*, *Firmicutes*, *Bacteroidetes*, *Chloroflexi*, *Pelotomaculum*, *Syntrophomonas*, *Smithllela*, *Syntrophus*, *Methanobacterium*, *Proteobacteria*, *Methanothermobacter*, *Methanobrevibacter*, *Methanoculleus*, *Methanospirillum*, etc. have been used (Patel et al., 2022). For example, the gram-positive *Bacillus thuringiensis* has been used to degrade naproxen and ibuprofen effectively (Marchlewicz et al., 2016). Likewise, the indigenous bacterial community containing *Bacillus* species and *Comamonas aquatica* has degraded ibuprofen (Fortunato et al., 2016). The potential of *Labrys portucalensis* F11 strain in removing drugs such as ofloxacin, ciprofloxacin, norfloxacin, and moxifloxacin has also been reported (Amorim et al., 2014; Carvalho et al., 2016). Besides, the species also eliminated diclofenac and carbamazepine (Bessa et al., 2017a, 2019; Moreira et al., 2018). Different bacteria are effective against specific contaminants. Brevibacterium species D4 was efficient in degrading diclofenac while carbamazepine was eliminated by *Starkeya* species C11 and *Rhizobium* species C12 (Bessa, Moreira, Tiritan, et al., 2017a, b).

Geobacillus species strain S-07 was effective in removing sulfamethazine at different efficiencies in co-metabolism with glucose (Pan et al., 2017). The biodegradation of sulfamethoxazole was demonstrated by three species namely *Ochrobactrum* species SA1, *Gordonia* species SCD14, and *Labrys* species SC11 (Mulla et al., 2018). Further, *Thermus* species strain C419 isolated from sludge degraded ciprofloxacin in sodium acetate as co-substrate (Pan et al., 2018). Apart from these, Zhang et al. (2013) showed that *Stenotrophomonas* species and *Pseudomonas* species were efficient in degrading paracetamol, one of the commonly used drugs for cold. Taken together, these studies highlight the importance and ability of diverse bacteria in the degradation of pharmaceutical wastes.

### Fungi

Fungi have also been demonstrated to be effective in the bioremediation of pharmaceutical contaminants. For instance, *Trametes versicolor* has eliminated drugs namely ibuprofen, carbamazepine, sulfamethoxazole, diclofenac, trimethoprim, clofibric acid, and naxopren found in wastewater (Alharbi et al., 2019; Marco-Urrea et al., 2009; Rodríguez-Rodríguez et al., 2010). Likewise, a study by Hachi et al. (2017) reported the active degradation of carbamazepine and acetaminophen by *Trametes hirsute*. Further, drugs reported in wastewater treatment plants such as citalopram, diclofenac, sulfamethoxazole, naproxen, ibuprofen, carbamazepine, fluoxetine, and diazepam were degraded by *Bjerkandera adusta*, *Phanerochaete chrysoporium*, and *Bjerkandera* species R1 (Rodarte-Morales et al., 2011). A consortium of fungi including *Trametes polyzona*, *Aspergillus niger*, *Rhizopus microspores*, *Trichoderma longibrachiatum*, and *Mucor circinelloides* have shown efficiency against ibuprofen, carbamazepine, and diclofenac (Kasonga et al., 2020). Additionally, research conducted by Rodarte-Morales and group (2012) has reported the elimination of diclofenac, naxopren, and ibuprofen by *Phanerochaete chrysoporium*. This fungus, along with *Irpex lacteus*, has also exhibited the ability to remove ibuprofen (Marco-Urrea et al., 2009)*.* In addition, *Pleurotus ostreatus* and *Trichoderma harzianum* converted carbamazepine and clarithromycin into non-toxic products (Buchicchio et al., 2016). Furthermore, studies have shown *Ganoderma lucidum* to degrade diclofenac, citalopram, valsartan, propranolol, carbamazepine, and ibuprofen effectively (Marco-Urrea et al., 2009; Mohd Hanafiah et al., 2024).

### Algae

At present, the use of microalgae in the treatment of pharmaceutical pollutants has been widely researched. Microalgae use diverse mechanistic approaches to remove pharmaceutical pollutants namely biosorption, bioaccumulation, and biodegradation. Biosorption involves the absorption of the drugs on the negatively charged polysaccharide-containing cell via mechanisms that include ion exchange reactions, surface complex formation, chelation, and microprecipitation. In bioaccumulation, the drugs are absorbed and accumulated within the cells through passive diffusion, passive facilitated diffusion, and active absorption. Biodegradation involves the catalytic breakdown of organic compounds through metabolic degradation and co-metabolism resulting in CO_2_ and water as byproducts (Gayosso-Morales et al., 2023).

For example, *Chlorella pyrenoidosa* together with UV algal technology effectively eliminated the antibiotic cefradine (Du et al., 2015). *Chlorella PY-ZU1*, a genetically modified microalgae mutant demonstrated 94% efficiency in removing ethinylestradiol (Cheng et al., 2018). Microalgae *Chlorella sorokiniana* has been utilized in clearing paracetamol detected in contaminated water (Escapa et al., 2017). Another study showed that green alga *Nannochloris* species efficiently removed triclosan while it failed to eliminate trimethoprim and sulfamethoxazole (Bai & Acharya, 2016). Both *Spirogyra* species and *Lemna* species showed diverse efficiencies in removing pharmaceutical wastes. A high removal rate was observed for acetaminophen; moderate levels for ethinylestradiol, ibuprofen, and propranolol; and poor removal efficiency for diclofenac, clofibric acid, and carbamazepine (Garcia-Rodríguez et al., 2015). Furthermore, *Scenedesmus obliquus* has been shown to eliminate levofloxacin (Xiong et al., 2017). These studies emphasize that the use of microorganisms in the remediation of pharmaceutical contaminants could be a promising technique.

## Biodegradation mechanisms of drugs

Microorganisms employ diverse degradation mechanisms to convert pharmaceutical pollutants into less toxic and more biodegradable compounds. The main degradation mechanisms observed include hydroxylation, oxidation, dehalogenation, ester cleavage, and ring fracture, depending on microbial enzyme activity (Jayasekara et al., 2025). For instance, *Labrys portucalensis* F11 primarily degrades diclofenac through hydroxylation, dechlorination, decarboxylation, and dehydrogenation, with additional pathways like methylation, HCl elimination, and sulfonation enhancing degradation (Moreira et al., 2018; Stylianou et al., 2018). Similarly, *Patulibacter medicamentivorans* degrades ibuprofen via hydroxylation, dehydrogenation, and oxidation, while *Pseudoxanthomonas* species DIN-3 facilitates further breakdown by removing carboxyl and hydroxyl groups (Salgado et al., 2020). Naproxen degradation involves hydroxylation, ester cleavage, and naphthalene ring fracture, followed by glucuronide group removal to enhance biodegradability (Navada & Kulal, 2019). Studies have shown that *Acinetobacter* species degrade sulfamethoxazole through hydroxylation and oxygenation, where oxygenase enzymes destabilize the aromatic ring for further breakdown (Wang & Wang, 2018). Chloramphenicol is degraded by *Trametes hirsuta* (laccase enzyme) via oxidation and dehalogenation, with laccase cleaving chlorine atoms and altering functional groups to reduce toxicity(Navada & Kulal, 2019). *Bosea* species As-6 degrades amoxicillin by hydroxylation and the beta-ketoadipate pathway, converting aromatic compounds into intermediates for the TCA cycle (Yan et al., 2022). Paracetamol, a widely used drug globally, is metabolized by *Pseudomonas moorei* KB4 through acetate release, hydroquinone formation, and hydroquinone cleavage (Żur et al., 2018). On the other hand, *Bacillus drentensis* strain S1 degrades paracetamol mainly via hydroquinone formation and hydroxylation, destabilizing the aromatic ring to facilitate breakdown (Chopra & Kumar, 2020). These microbial degradation pathways play a crucial role in reducing pharmaceutical pollution, making contaminants less toxic and more environmentally friendly. Table 2 lists some of their microorganism and the degradation mechanism.

**Table 2:** Biodegradation mechanisms of pharmaceutical drugs

Drugs	Species	Mechanism of degradation	References
Diclofenac	*Labrys portucalensis* F11	Hydroxylation, dechlorination, decarboxylation, dehydrogenation, methylation, HCl elimination, sulfonation	(Moreira et al., 2018; Stylianou et al., 2018)
Ibuprofen	*Patulibacter medicamentivorans*	Hydroxylation, dehydrogenation, oxidation	(Salgado et al., 2020)
	*Pseudoxanthomonas* sp. DIN-3	Hydroxylation, carboxyl and hydroxyl group loss, oxidation	(Salgado et al., 2020)
Naproxen	*Pseudoxanthomonas* sp. DIN-3	Hydroxylation, ester group cleavage, naphthalene ring fracture, hydroxyl and glucuronide group loss	(Lu et al., 2019)
Sulfamethoxazole	*Acinetobacter* species	Hydroxylation, oxygenation	(Wang & Wang, 2018)
Chloramphenicol	*Trametes hirsuta* (laccase enzyme)	Oxidation, dehalogenation	(Navada & Kulal, 2019)
Amoxicillin	*Bosea* species As-6	Hydroxylation, beta-ketoadipate pathway oxidation	(Yan et al., 2022)
Paracetamol	*Pseudomonas moorei* KB4	Acetate release, hydroquinone formation, hydroquinone cleavage	(Żur et al., 2018)
	*Bacillus drentensis* strain S1	Hydroquinone formation, hydroxylation	(Chopra & Kumar, 2020)

## Challenges of bioremediation

Although studies have demonstrated bioremediation to be a potential approach in the treatment of pharmaceutical pollutants, there exist a lot of challenges that require attention. The primary challenge is to minimize the time requirements in treating these contaminants which depends on factors such as the type of microorganisms used, nutrient amendments, and environmental growth conditions. Treating the sites comprising diverse pharmaceutical contaminants becomes the next challenge as different microorganisms and bioremediation techniques are to be employed in degrading them. Apart from this, using bioremediation techniques for sites with high concentrations of pharmaceutical contaminants is also a major limitation as the conditions may not be favorable for bacterial activity. Apart from this, proper handling of bioengineered microorganisms becomes crucial as well (Saravanan et al., 2024).

## Sustainable green approach

In addition to utilizing bioremediation approaches to manage pharmaceutical waste, “green pharmacy” approaches can be employed to enhance product sustainability. Manufacturing companies should ensure that pharmaceuticals manufactured are not persistent in the environment. Novel methods should be employed to develop pharmaceutical products that remain stable in the patient’s body but degrade after excretion. Furthermore, for substances that meet the PBT (persistent, bioaccumulative, and toxic) criteria, minimizing the exposure should be a primary objective. Additional approaches should focus on avoiding the use of molecular moieties that pose potential risk to environment. Implementing this green pharmacy approach presents great challenges, necessitating collaborative initiatives between pharmaceutical industries and academia to adapt to green practices in the pharmaceutical development process (Moermond et al., 2022; Srivastava et al., 2022).

Furthermore, an integral aspect of green pharmacy and sustainable pharmaceutical manufacturing is effective sewage treatment before discharge into the environment. Advanced wastewater treatment technologies, such as biodegradation, membrane filtration, and advanced oxidation processes, play a critical role in removing pharmaceutical residues from wastewater. Implementing these strategies ensures that drug residues are efficiently broken down, preventing their accumulation in aquatic ecosystems. By incorporating sustainable waste management techniques, the pharmaceutical industry can minimize the environmental footprint of drug manufacturing and disposal, contributing to a safer and more eco-friendly approach (Kumar et al., 2023; Ullah et al., 2025).

## Conclusion

With lifestyle changes, the use of pharmaceutical products has become inevitable. Consequently, pharmaceutical compounds have become a global threat due to their discharge into the environment, causing various health issues in humans and animals even at minute concentrations. While physical and chemical bioremediation are commonly used to treat various environmental pollutants, they are expensive and may not be a suitable option for pharmaceutical contaminants. As an alternative approach, bioremediation has gained attention in recent times wherein microorganisms are used to treat the pollutants. This review has discussed the various microorganisms that have been employed to degrade widely reported pharmaceutical pollutants in the environment like ibuprofen, diclofenac, sulfamethoxazole, etc. Further, different bioremediation techniques investigated to treat these contaminants and found to be effective in laboratory scale has been explored. However, there exists limitations such as time consumption, control over microbial activity, high pollutant concentrations and handling of bioengineered microorganism that need to be addressed. Beyond finding solutions to remediate pharmaceutical pollutants, focusing on sustainable green pharmacy approaches will help reduce the amount of pharmaceutical pollutants in the environment.

## Data Availability

No datasets were generated or analysed during the current study.
